# ER Stress-Mediated Impairment of Hepatic Lipid Export Drives Steatosis in AKI-Induced Remote Liver Injury

**DOI:** 10.21203/rs.3.rs-9409706/v1

**Published:** 2026-05-19

**Authors:** Minghua Li, Runze Ni, Kristof Williams, Miriame Melaika, Lucas Eli Sun, Liying Fu, Vijay Subramanian, Kiran Dhanireddy, Ruisheng Liu

**Affiliations:** University of South Florida (USF); University of South Florida (USF); University of South Florida (USF); University of South Florida (USF); University of South Florida (USF); Mayo Clinic; Tampa General Hospital; Tampa General Hospital; University of South Florida (USF)

**Keywords:** AKI, Remote organ injury, Hepatic steatosis, ER Stress, VLDL secretion, clinical validation, lipid dissociation

## Abstract

**Background:**

Acute kidney injury (AKI) frequently causes remote organ injury including hepatic steatosis, yet whether lipid accumulation reflects increased synthesis or impaired clearance has not been resolved.

**Methods:**

We used a murine ischemia-reperfusion AKI model. Unbiased liver proteomics was performed at 24 hours after reperfusion, and dysregulated pathways were identified by Gene Set Enrichment Analysis. Results were validated by Western blotting, qPCR, and immunohistochemistry. These findings were complemented by retrospective analysis of two ICU databases (MIMIC-IV and eICU-CRD).

**Results:**

AKI significantly increased serum ALT and AST and induced hepatic lipid accumulation. Proteomic analysis revealed that key lipogenic enzymes (Scd1, Fasn, Acly, Acaca) were uniformly suppressed rather than upregulated. Proteins essential for VLDL assembly (ApoB, MTTP, ApoE) were significantly downregulated. Plasma triglycerides were decreased while liver triglycerides were increased, consistent with impaired hepatic lipid export. As in renal tubular cells, AKI also disrupted ER protein folding homeostasis in the liver, triggering ER stress. This was evidenced by upregulated levels of the ER chaperone GRP78, increased XBP1 splicing indicative of UPR activation, and elevated expression of the ER stress-induced pro-apoptotic transcription factor CHOP, suggesting that prolonged ER stress may also promote hepatocyte cell death. TLR4/MyD88 signaling was activated, yet inflammatory cytokines were paradoxically reduced, accompanied by Kupffer cell depletion (decreased F4/80) and monocyte infiltration (increased CD68). In 6,996 propensity-matched ICU patients (MIMIC-IV), AKI independently increased the risk of clinically significant liver injury 4-fold (adjusted OR = 4.41). Analysis of 22,727 patients across 208 hospitals (eICU-CRD) identified a lipid dissociation pattern: elevated triglycerides alongside decreased total cholesterol, HDL, and LDL, with dose-dependent scaling across KDIGO stages.

**Conclusions:**

These data support ER stress-mediated impairment of VLDL export as a primary driver of AKI-induced hepatic steatosis. Clinical validation across two independent ICU databases identifies a dual metabolic insult: enhanced peripheral lipid delivery compounds impaired hepatic export, amplifying hepatic lipid retention. ER stress and lipid export machinery represent potential therapeutic targets for AKI-associated liver injury.

## Background

Acute kidney injury (AKI) extends far beyond kidney dysfunction. In critically ill patients, AKI-associated mortality largely derives from distant organ injury—a phenomenon termed kidney-organ crosstalk^1,2^. Due to the anatomical proximity and shared venous circulation, liver represents a particularly vulnerable target. While inflammatory cascades and oxidative stress have long been implicated in AKI-induced hepatic injury^1,3^, the metabolic consequences remain surprisingly under-explored. This gap is significant. As the body's central metabolic hub, the liver orchestrates lipid homeostasis, protein synthesis, and detoxification; disruption of these functions during AKI may compound systemic deterioration and worsen clinical outcomes. Clinical observations in ICU populations have linked AKI to hepatic dysfunction and serum lipid derangements, yet the metabolic basis of these associations has not been systematically defined^1,3,4^. Bridging this gap requires integration of mechanistic animal models with clinical cohort analysis.

The pathological accumulation of triglycerides within hepatocytes causes hepatic steatosis. And, among the hepatic manifestations of AKI, steatosis emerged as a consistent finding in experimental models^5,6^. Hepatic triglyceride homeostasis reflects a precise balance between lipid acquisition through fatty acid uptake and de novo lipogenesis, and disposal through β-oxidation and VLDL-mediated export^7–9^. Steatosis develops when this equilibrium shifts toward accumulation: either through increased fatty acid influx (from dietary sources or enhanced adipose lipolysis), upregulated lipogenesis, impaired oxidation, or defective export. AKI, however, represents none of these classical scenarios. It is neither a fed state nor one of caloric surplus; rather, it induces profound metabolic stress and catabolism. In this catabolic state, the liver should be exporting lipids to meet systemic energy demands, not retaining them. Paradoxically, triglycerides accumulate within hepatocytes. How this occurs has not been clearly defined. Key enzymes governing fatty acid synthesis including stearoyl-CoA desaturase 1 (Scd1) and fatty acid synthase (Fasn) showed marked downregulation. Simultaneously, fatty acid oxidation capacity appeared diminished. These findings suggest the liver is neither synthesizing excess fat nor failing to burn existing stores at appreciably altered rates. And this led us to reasonable hypothesis that lipid is being under-exported.

The endoplasmic reticulum (ER) provides a plausible mechanistic link. This ER serves as the assembly site for very-low-density lipoproteins (VLDL), the primary vehicle for hepatic triglyceride export. Proper VLDL formation requires coordinated action of apolipoprotein B (ApoB) and microsomal triglyceride transfer protein (MTTP). And both are highly sensitive to disruptions in ER protein folding^10,11^. During AKI, circulating damage-associated molecular patterns (DAMPs) activate hepatic Toll-like receptor 4 (TLR4) and its adaptor MyD88^12,13^. This signaling cascade could trigger the unfolded protein response (UPR), evidenced by elevated expression of ER stress markers such as GRP78, CHOP, and spliced XBP1^14^. Prolonged ER stress overwhelms protein folding capacity, promoting degradation of nascent ApoB and impairing VLDL assembly. As a result, lipid that would normally be exported accumulate within hepatocytes.

Here, we present proteomic and molecular evidence supporting an ER stress-mediated impairment of hepatic lipid export as a primary driver of AKI-induced steatosis. In classical steatosis models, hepatic fat accumulation reflects a net imbalance favoring lipid acquisition over disposal. Our data suggest a distinct paradigm in AKI. Despite suppressed lipogenesis, triglycerides accumulate, pointing to impaired export as the primary driver. This mechanistic distinction might reveal new strategies for preventing hepatic dysfunction during acute kidney injury. To assess clinical relevance, we additionally analyzed two large ICU databases (MIMIC-IV and eICU-CRD), examining hepatic injury patterns and lipid profiles in patients with and without AKI.

## Methods

### Animals

Male C57BL/6J mice (10–12 weeks old, 25 ± 3 g) were purchased from The Jackson Laboratory (Bar Harbor, ME; stock #000664) and acclimatized for at least one week before experiments. Mice were group-housed in a temperature- and humidity-controlled facility maintained on a 12-hour light/dark cycle, with ad libitum access to standard rodent chow and water. All animal studies were approved by the Institutional Animal Care and Use Committee at the University of South Florida, Morsani College of Medicine (protocol 12005R) and conducted in accordance with the National Institutes of Health Guide for the Care and Use of Laboratory Animals. Anesthesia was induced and maintained with inhaled isoflurane (2–3% in oxygen) throughout surgical procedures. At the 24-hour endpoint, mice were placed under deep isoflurane anesthesia. Following confirmation of deep anesthesia, mice were euthanized by exsanguination via cardiac puncture, followed immediately by the harvest of kidney and liver tissues.

## Renal Ischemia-Reperfusion Injury Model (IRI-AKI)

IRI-AKI was induced by bilateral renal ischemia-reperfusion based on similar methods as we previously described^15–17^. Male C57BL/6 mice (10–12 weeks, 25 ± 3 g) underwent bilateral renal pedicle clamping for 30 min while body temperature was maintained at 36.8–37.2°C. Following reperfusion, mice were allowed to recover with free access to food and water and were sacrificed 24 h post-injury.

## Retrospective Clinical Cohort Analysis

We analyzed data from the Medical Information Mart for Intensive Care IV database (MIMIC-IV, version 3.1), which contains over 94,000 ICU admissions at Beth Israel Deaconess Medical Center between 2008 and 2022^18–20^. AKI was defined by Kidney Disease: Improving Global Outcomes (KDIGO) serum creatinine criteria, with baseline creatinine taken as the lowest value within seven days of ICU admission. Patients were excluded for pre-existing liver disease (ICD-9 571.x; ICD-10 K70–K77), chronic kidney disease stage 3 or higher, prior liver transplantation, ICU stay shorter than 24 hours, missing baseline laboratory values, or baseline aminotransferase levels exceedingly twice the upper limit of normal. Non-AKI ICU patients meeting the same exclusion criteria served as the control pool. Propensity score matching was performed at a 1:1 ratio using nearest-neighbor matching (caliper = 0.2 standard deviations of the logit-transformed propensity score), with covariates including age, sex, non-renal Sequential Organ Failure Assessment (SOFA) score, Sepsis-3 status, diabetes, vasopressor use, and admission category. Peak liver markers (ALT, AST, total bilirubin, alkaline phosphatase) and albumin nadir were extracted within 72 hours of AKI onset or the matched reference time for controls. Serum triglyceride levels were also extracted for an exploratory lipid analysis.

We independently analyzed data from the eICU Collaborative Research Database (eICU-CRD, version 2.0), a multi-center database comprising 200,859 ICU admissions from 208 hospitals across the United States (2014–2015)^21,22^. Adult patients (age ≥ 18) with at least one serum creatinine measurement and an ICU length of stay of 24 hours or more were eligible. Patients with pre-existing end-stage renal disease, chronic dialysis, or liver cirrhosis were excluded. AKI was defined using KDIGO creatinine criteria, with baseline creatinine taken as the first value measured within 24 hours of ICU admission. Serum lipid measurements (triglycerides, total cholesterol, HDL cholesterol, LDL cholesterol) were extracted from the laboratory table using timestamped offsets from ICU admission.

Between-group comparisons used Mann-Whitney U tests given non-normal distributions, and dose-response relationships across KDIGO stages were assessed by Kruskal-Wallis tests. For the MIMIC-IV cohort, multivariable logistic regression estimated the adjusted odds of clinically significant liver injury (defined as ALT > 80 U/L), with covariates including age, sex, non-renal SOFA score, sepsis status, vasopressor use, diabetes, statin use, acetaminophen exposure, and mechanical ventilation. For the eICU-CRD cohort, multivariable linear regression of log-transformed lipid values adjusted for age, sex, APACHE IV score, body mass index, and diabetes status. Temporal trajectories in both cohorts were modeled using linear mixed-effects models with random intercepts. Subgroup analyses in eICU-CRD examined consistency across sepsis status, admission category, sex, and diabetes status. Cross-database concordance was assessed by comparing the direction and magnitude of lipid changes between the two cohorts. Both databases were accessed through PhysioNet with signed data use agreements; institutional review board approval was waived as the data are fully de-identified. Clinical analyses were performed in Python 3 using scipy, statsmodels, and tableone. Full details, including cohort derivation flowcharts and covariate balance assessment, are provided in the Supplementary Methods.

### Statistical Analysis

Data are expressed as mean ± SEM. Statistical comparisons between Sham and AKI groups were performed using unpaired two-tailed Student's t-test. For proteomics data, differential protein abundance was assessed using DEqMS, and P-values were adjusted for multiple testing using the Benjamini-Hochberg method. A P-value < 0.05 was considered statistically significant. Statistical analyses were performed using GraphPad Prism 10 (GraphPad Software, San Diego, CA) and R (v4.5.2).

Detailed protocols for standard biochemical assays (Western blotting, PCR, etc.) are in the Supplementary Methods

## Results

### AKI induces hepatic injury and lipid accumulation

**Figure 1:**
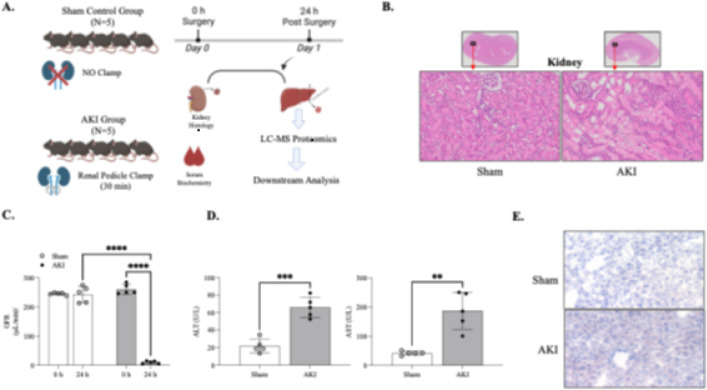
AKI Induces Remote Hepatic Injury and Lipid Accumulation (A) Experimental design schematic. Male C57BL/6 mice (10–12 weeks) underwent bilateral renal pedicle clamping for 30 minutes (AKI group, n=5) or sham surgery without clamping (Sham group, n=5). Liver tissue was harvested 24 hours post-reperfusion for LC-MS proteomics and complementary molecular analyses. (B) Representative hematoxylin and eosin (H&E)-stained kidney sections from Sham (CON) and AKI mice. AKI kidneys display characteristic ischemia-reperfusion injury features including tubular dilation, epithelial flattening, and loss of brush border integrity. Insets show higher magnification of boxed regions. Scale bars: 100 μm (low magnification), 50 μm (high magnification). (C) Glomerular filtration rate (GFR) measured at baseline (0 h) and 24 hours post-surgery. AKI mice demonstrate near-complete loss of filtration capacity at 24 hours compared to Sham controls. Data are mean ± SEM; n=5 per group. ****p < 0.0001 by two-way ANOVA with Sidak’s multiple comparisons test; ns, not significant. (D) Serum alanine aminotransferase (ALT, left) and aspartate aminotransferase (AST, right) levels indicating hepatocellular injury. Both markers are significantly elevated in AKI mice. Data are mean ± SEM; n=5 per group. ***p < 0.001, **p < 0.01 by unpaired two-tailed Student’s t-test. (E) Representative Oil Red O staining of liver sections demonstrating hepatic lipid accumulation. AKI livers display pronounced lipid droplet deposition within hepatocytes compared to minimal staining in Sham controls. Scale bar: 50 μm.

To investigate the remote hepatic effects of acute kidney injury, we established a murine ischemia-reperfusion AKI model using bilateral renal pedicle clamping (30 minutes) and assessed both renal and hepatic outcomes at 24 hours post-injury (Fig. 1A). Liver tissue was subsequently analyzed using quantitative proteomics complemented by targeted molecular validation.

Successful AKI induction was confirmed by histological and functional assessments. Kidney sections from the sham group displayed normal baseline architecture. Glomeruli remained entirely intact. Proximal tubules exhibited the expected tall cuboidal epithelium and prominent eosinophilic brush borders, completely devoid of luminal debris. Tissues from the AKI group revealed profound tubulointerstitial disruption. Widespread loss of the apical brush border occurred (Fig. 1B). GFR measurements demonstrated profound renal dysfunction: while baseline values remained comparable between groups at 0 hours, AKI mice exhibited near-complete loss of filtration capacity at 24 hours (10.5 ± 4.6 μL/min vs. 243.7 ± 27.9 μL/min in sham, p < 0.0001; Fig. 1C). Molecular markers of tubular injury corroborated these findings. Plasma creatinine level analysis showed marked upregulation in AKI mice plasma (S. Figure 1A).

Concurrent hepatic dysfunction was evident in AKI animals. Serum ALT increased approximately 3-fold compared to controls (66.1 ± 11.8 vs. 21.7 ± 7.9 U/L, p < 0.001; Fig. 1D). AST levels were also significantly elevated following AKI (187.5 ± 63.6 vs. 42.2 ± 6.9 U/L, p < 0.01), though individual variability was noted (Fig. 1D). To assess hepatic lipid accumulation, we performed Oil Red O staining on liver sections. AKI livers displayed pronounced lipid droplet deposition within hepatocytes, contrasting sharply with minimal staining in sham controls (Fig. 1E). Quantification revealed a substantial increase in Oil Red O-positive area following AKI (p < 0.05). These findings establish that AKI triggers remote liver injury characterized by hepatocyte dysfunction and steatosis.

## LC-MS Proteomic Profiling Identifies ER Stress Activation and Global Metabolic Suppression

**Figure 2:**
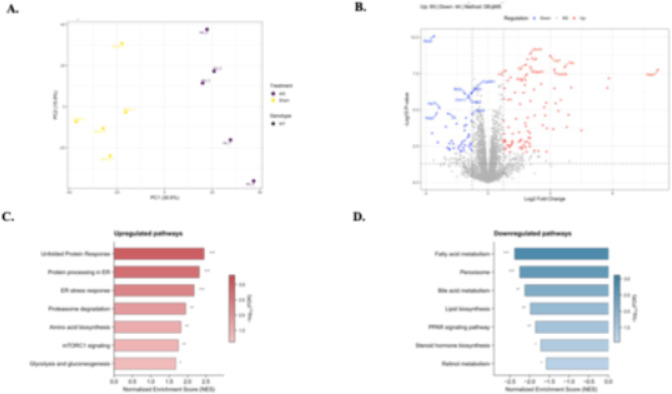
Hepatic Proteomics Reveals ER Stress Activation and Global Metabolic Suppression Following AKI (A) Principal component analysis (PCA) of liver proteomes showing distinct clustering between Sham (yellow) and AKI (purple) groups. PC1 accounts for 30.6% and PC2 for 15.9% of total variance, indicating a reproducible and distinct hepatic proteomic signature induced by AKI. n=5 per group. (B) Volcano plot depicting differentially expressed proteins (DEPs) between AKI and Sham livers. Analysis using DEqMS identified 129 significantly altered proteins (85 upregulated, red; 44 downregulated, blue) with thresholds of adjusted p < 0.05 and |log_2_ fold-change| > 1. Representative proteins are labeled. NS, not significant (gray). (C) Gene set enrichment analysis (GSEA) of upregulated pathways in AKI livers. Bar length represents normalized enrichment score (NES); color intensity indicates statistical significance (−log_10_FDR). The unfolded protein response (UPR), protein processing in ER, and ER stress response pathways show robust enrichment. *p < 0.05, **p < 0.01, ***p < 0.001. (D) GSEA of downregulated pathways demonstrating global suppression of hepatic metabolic functions. Fatty acid metabolism, peroxisome, bile acid metabolism, lipid biosynthesis, and PPAR signaling pathways are uniformly suppressed. Bar length represents NES; color intensity indicates −log_10_FDR. *p < 0.05, **p < 0.01, ***p < 0.001.

To identify molecular drivers of AKI-induced hepatic dysfunction, we performed label-free quantitative proteomics on liver tissue harvested 24 hours post-injury. Principal component analysis revealed robust separation between experimental groups, with PC1 accounting for 30.6% and PC2 for 15.9% of total variance (Fig. 2A). This clear clustering indicates that AKI induces a distinct and reproducible hepatic proteomic signature. Differential expression analysis using DEqMS identified 129 significantly altered proteins—85 upregulated and 44 downregulated—applying thresholds of adjusted p < 0.05 and absolute log_2_ fold-change > 1 (Fig. 2B). The complete list of differentially expressed proteins is provided in Table S1.

Gene set enrichment analysis yielded a striking pattern. Among upregulated pathways, the unfolded protein response emerged as the most significantly enriched (NES = 2.08, FDR < 0.001), followed closely by protein processing in the endoplasmic reticulum and ER stress response (Fig. 2C). Additional enrichment was observed in proteasome degradation, amino acid biosynthesis, and mTORC1 signaling—consistent with a coordinated cellular response to proteotoxic stress (Table S2).

**Figure 3:**
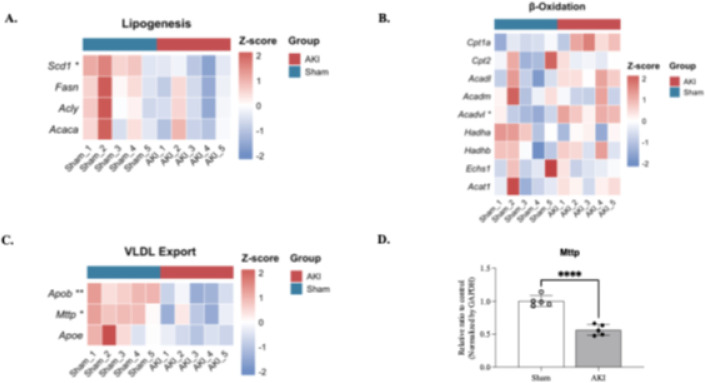
Paradoxical Lipid Accumulation Despite Suppressed Lipogenesis Points to VLDL Export Failure (A) Heatmap displaying Z-score normalized protein abundance of key lipogenic enzymes across individual samples. Scd1, Fasn, Acly, and Acaca are uniformly downregulated in AKI livers, arguing against enhanced lipid synthesis as a driver of steatosis. Asterisks denote statistical significance (*adj. p < 0.05). (B) Heatmap of β-oxidation pathway proteins showing broad suppression in AKI livers. Mitochondrial fatty acid import (Cpt1a, Cpt2) and β-oxidation spiral enzymes (Acadl, Acadm, Acadvl, Hadha, Hadhb, Echs1, Acat1) demonstrate coordinate downregulation. Z-scores calculated across all samples. *adj. p < 0.05. (C) Heatmap of VLDL export machinery proteins. ApoB, MTTP, and ApoE—essential components of VLDL assembly and secretion—are markedly reduced in AKI livers. Statistical significance indicated by asterisks on protein names (**adj. p < 0.01, ***adj. p < 0.001). (D) Quantitative RT-PCR validation of Mttp transcript levels. Mttp expression is significantly reduced in AKI compared to Sham livers, confirming impaired VLDL assembly machinery at the transcript level. Expression normalized to GAPDH and presented relative to Sham controls. Data are mean ± SEM; n=5 per group. ****p < 0.0001 by unpaired two-tailed Student’s t-test.

The downregulated pathways painted an equally coherent picture. Fatty acid metabolism exhibited the strongest suppression (NES = − 2.03, FDR < 0.001), with peroxisomal pathways similarly diminished (NES = − 2.26, FDR < 0.001; Fig. 2D). Bile acid metabolism, lipid biosynthesis, and PPAR signaling—central regulators of hepatic lipid handling—were uniformly suppressed. Xenobiotic metabolism also showed marked downregulation (NES = − 2.01, FDR < 0.001), indicating broad impairment of hepatic metabolic and detoxification functions (S. Figure 3A-B; Table S2). KEGG pathway enrichment (S. Figure 3B) corroborated these findings, with peroxisome, fatty acid metabolism, and xenobiotic/drug metabolism among the top suppressed pathways^23,24^. This pattern of ER stress activation concurrent with global metabolic suppression prompted closer examination of specific proteins within the lipid synthesis and export pathways.

## Impaired VLDL Export Machinery Underlies Hepatic Lipid Retention

The observation of hepatic steatosis alongside suppressed lipid metabolism pathways presented an apparent paradox. To resolve this contradiction, we examined individual protein changes across distinct arms of hepatic lipid handling (Table 2).

Lipogenic enzymes were uniformly downregulated. Scd1, the rate-limiting enzyme for monounsaturated fatty acid synthesis, showed significant suppression (log_2_FC = − 1.59, adj. p = 0.031). Fasn, Acly, and Acaca—additional key mediators of de novo lipogenesis—demonstrated concordant reductions, though with varying statistical significance (Fig. 3A). These findings argue against enhanced lipid synthesis as a driver of steatosis.

β-oxidation machinery demonstrated broad suppression as well. Cpt1a, the rate-limiting enzyme for mitochondrial fatty acid import, was decreased alongside Cpt2 (Fig. 3B). The acyl-CoA dehydrogenases (Acadl, Acadm, Acadvl) and enzymes of the β-oxidation spiral—including Hadha, Hadhb, Echs1, and Acat1—showed coordinate downregulation. Transcript-level analysis confirmed impaired fatty acid handling: Fabp1, encoding liver fatty acid-binding protein, was significantly reduced in AKI livers (0.54 ± 0.08-fold, p < 0.05), while Acox1 expression showed a compensatory increase (1.75 ± 0.20-fold, p < 0.05; S. Figure 2C).

A different picture emerged when examining lipid export machinery. ApoB, the structural apolipoprotein essential for VLDL assembly, was significantly reduced (log_2_FC = − 0.23, adj. p = 0.005). MTTP, the microsomal triglyceride transfer protein required for ApoB lipidation, showed comparable suppression (log_2_FC = − 0.25, adj. p = 0.022). ApoE, which facilitates VLDL secretion and hepatic lipid clearance, demonstrated a strong downward trend (log_2_FC = − 0.56, adj. p = 0.072; Fig. 3C, Table 1). Quantitative RT-PCR confirmed significant reduction of Mttp transcript levels in AKI livers (p < 0.0001; Fig. 3D). These data suggest that while both lipid synthesis and oxidation are suppressed, the magnitude of export impairment exceeds synthesis reduction, resulting in net hepatic lipid retention.

### ER Stress Is Robustly Activated in AKI Livers

**Figure 4:**
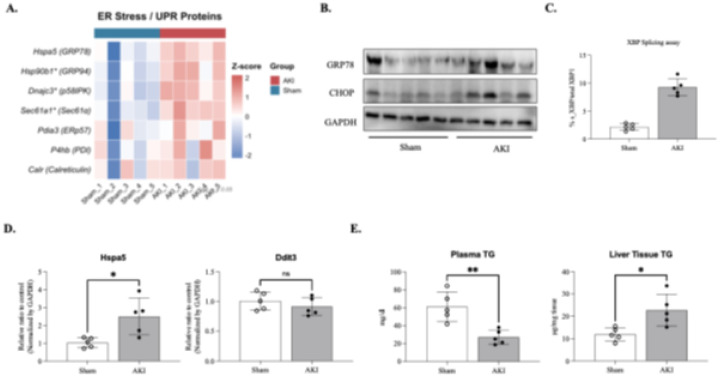
Robust Activation of the Unfolded Protein Response in AKI Livers (A) Heatmap of ER stress and UPR-associated proteins identified by proteomics. Hspa5 (GRP78), Hsp90b1 (GRP94), Dnajc3 (p58IPK), and Sec61a1 show elevated abundance in AKI livers, with additional chaperones Pdia3 (ERp57), P4hb (PDI), and Calr (Calreticulin) displaying concordant trends. Z-scores calculated across all samples; asterisks indicate adj. p < 0.05. (B) Western blot analysis of ER stress markers GRP78/BiP and CHOP. Both proteins are significantly elevated in AKI livers compared to Sham controls. Representative blots are shown with GAPDH as loading control. All lanes shown are contiguous from single membranes; no splicing was performed. (C) XBP1 splicing assay measuring the proportion of spliced XBP1 (sXBP1) relative to total XBP1 transcript in liver tissue. AKI livers showed a marked increase in sXBP1/total XBP1 ratio compared to Sham controls, indicating activation of the IRE1α arm of the unfolded protein response. Data are presented as mean ± SD; n = 5 per group. (D) Quantitative RT-PCR validation of ER stress markers. Hspa5 (encoding GRP78) transcript levels are significantly upregulated in AKI livers (*p < 0.05), while Ddit3 (encoding CHOP) shows an upward trend that does not reach statistical significance (ns). Expression normalized to GAPDH and presented relative to Sham controls. Data are mean ± SEM; n=5 per group. (E) Triglyceride measurements in plasma and liver tissue. Left: Plasma triglyceride (TG) levels are significantly reduced in AKI mice compared to Sham controls, consistent with impaired VLDL-mediated lipid export. Right: Liver tissue TG content is significantly elevated in AKI livers, confirming intrahepatic lipid retention. Data are mean ± SEM; n=5 per group. **p < 0.01 (plasma TG), *p < 0.05 (liver TG) by unpaired two-tailed Student’s t-test.

Given that ER stress can directly impair ApoB folding and VLDL assembly, we validated UPR activation through orthogonal approaches. Proteomics identified upregulation of multiple ER chaperones and stress response proteins (Fig. 4A). Hspa5/GRP78, the master regulator of the UPR, showed elevated abundance (log_2_FC = + 0.36, adj. p = 0.052), as did Hsp90b1/GRP94 (log_2_FC = + 0.42, adj. p = 0.036), Dnajc3/p58IPK (log_2_FC = + 0.43, adj. p = 0.018), and Sec61a1 (log_2_FC = + 0.36, adj. p = 0.021; Table 1). Several additional ER-resident proteins showed concordant trends, including Pdia3/ERp57, P4hb/PDI, and calreticulin.

Western blot analysis confirmed elevated protein levels of key UPR markers. GRP78/BiP increased significantly in AKI livers compared to sham controls (Fig. 4B). CHOP, a pro-apoptotic transcription factor induced during prolonged ER stress, was similarly elevated (Fig. 4B). These protein-level changes were corroborated at the transcript level: qPCR demonstrated significant upregulation of Hspa5 in AKI livers, while Ddit3/CHOP showed an upward trend that did not reach statistical significance (Fig. 4D).

To assess functional engagement of the IRE1α-XBP1 branch, we quantified XBP1 splicing by qPCR. Under ER stress, activated IRE1α cleaves XBP1 mRNA to generate the spliced isoform (XBP1s), providing a direct readout of UPR activation. Spliced XBP1 transcripts were markedly increased in AKI livers (Fig. 4C). Collectively, these findings demonstrate robust activation of the hepatic UPR following AKI, involving both the IRE1α-XBP1 and PERK-CHOP signaling branches. To directly assess the functional consequence of impaired VLDL export, we measured triglyceride levels in both plasma and liver tissue. Plasma triglyceride concentrations were significantly reduced in AKI mice compared to sham controls (p < 0.01; Fig. 4E, left), consistent with diminished hepatic VLDL secretion. Conversely, liver tissue triglyceride content was significantly elevated in AKI livers (p < 0.05; Fig. 4D, right), confirming intrahepatic lipid retention. This reciprocal pattern—decreased circulating triglycerides coupled with increased hepatic triglyceride accumulation—provides direct evidence that impaired VLDL-mediated export, rather than enhanced lipogenesis, drives AKI-induced hepatic steatosis.

### TLR4 Signaling Is Engaged Without Classical NF-κB-Dependent Inflammation

**Figure 5:**
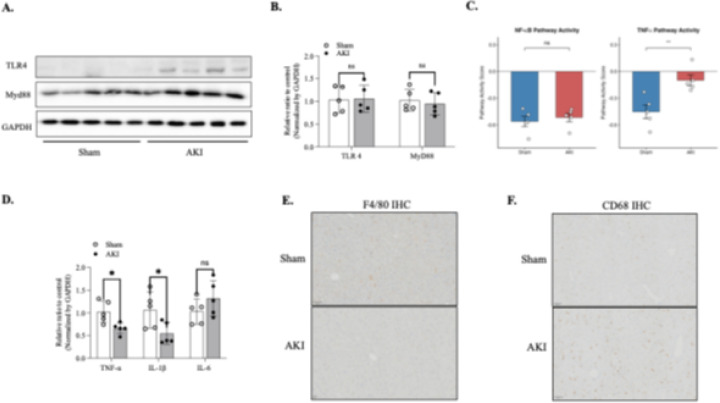
TLR4/MyD88 Activation Without Classical NF-κB-Dependent Inflammation (A) Representative Western blots of TLR4 and MyD88 protein levels in liver tissue. GAPDH serves as loading control. All lanes shown are contiguous from single membranes; no splicing was performed. (B) Densitometric quantification of TLR4 and MyD88 Western blots. While modest elevations are observed, changes do not reach statistical significance at the protein level. Data are mean ± SEM; n=5 per group. ns, not significant by unpaired two-tailed Student’s t-test. (C) PROGENy pathway activity analysis derived from proteomics data. NF-κB pathway activity remains unchanged between groups (left), while TNF-α pathway activity is paradoxically elevated in AKI livers despite suppressed cytokine expression (right). This dissociation suggests post-transcriptional regulation of inflammatory output. Data are mean ± SEM; n=5 per group. ns, not significant; **p < 0.01 by unpaired two-tailed Student’s t-test. (D) Quantitative RT-PCR analysis of pro-inflammatory cytokines. TNF-α and IL-1β transcripts are significantly decreased in AKI livers; IL-6 shows a non-significant trend toward reduction. Expression normalized to GAPDH and presented relative to Sham controls. Data are mean ± SEM; n=5 per group. *p < 0.05 by unpaired two-tailed Student’s t-test; ns, not significant. (E) Representative F4/80 immunohistochemistry demonstrating reduced Kupffer cell populations in AKI livers. Positive cells appear brown. Scale bar: 50 μm.(F) Representative CD68 immunohistochemistry showing increased monocyte-derived macrophage infiltration in AKI livers. Positive cells appear brown. Scale bar: 50 μm.

We next investigated upstream signaling events linking AKI to hepatic ER stress. TLR4 and its downstream adaptor MyD88 were examined at the protein level by Western blot (Fig. 5A–B). While receptor protein levels showed modest elevation that did not reach statistical significance (Fig. 5B), transcript-level analysis of the TLR4 co-receptor CD14 revealed significant upregulation in AKI livers (2.73 ± 0.58-fold, p < 0.05; S. Figure 2A). MD-2, another TLR4 accessory protein, showed no significant change (S. Figure 2A). These findings suggest priming of the TLR4 signaling complex.

To assess whether receptor-level engagement propagated through canonical inflammatory cascades, we performed PROGENy pathway activity analysis on our proteomics dataset. Despite evidence of TLR4 complex activation, NF-κB pathway activity remained unchanged between AKI and sham livers (Fig. 5C). This indicates that classical TLR4-mediated inflammatory signaling is not engaged at the transcription factor level.

Consistent with suppressed NF-κB activity, pro-inflammatory cytokine expression was paradoxically reduced. TNF-α mRNA decreased significantly (0.66 ± 0.12-fold, p < 0.05), as did IL-1β (0.55 ± 0.24-fold, p < 0.05; Fig. 5D). IL-6 showed a non-significant trend toward reduction (Fig. 5D). Cytokine array analysis corroborated these findings at the protein level (S. Figure 1B, C).

Intriguingly, PROGENy analysis revealed a dissociation between TNF-α pathway activity and cytokine expression. While TNF-α transcript and protein levels were suppressed, TNF-α pathway activity—reflecting downstream signaling competence—was paradoxically elevated in AKI livers (p < 0.01; Fig. 5C). This discordance suggests post-transcriptional mechanisms account for reduced cytokine output. One plausible explanation involves regulated IRE1-dependent decay (RIDD), whereby activated IRE1α degrades select mRNAs including inflammatory cytokine transcripts.

### Macrophage Population Dynamics Accompany the Inflammatory Paradox

Immunohistochemical analysis offered additional insight into the dissociation between receptor activation and cytokine production. F4/80-positive cells, representing resident Kupffer cells, were significantly decreased in AKI livers (Fig. 5E). Transcript analysis confirmed this reduction (0.67 ± 0.11-fold, p < 0.05; S. Figure 2B). Conversely, CD68-positive cells—likely representing infiltrating monocyte-derived macrophages—increased substantially (Fig. 5F), with qPCR showing elevated CD68 expression (1.58 ± 0.18-fold, p < 0.05; S. Figure 2B). CD163, a marker associated with alternatively activated macrophages, was also significantly upregulated (2.15 ± 0.25-fold, p < 0.05; S. Figure 2B), suggesting the infiltrating population may exhibit an anti-inflammatory phenotype.

This phenotypic shift—characterized by Kupffer cell depletion, monocyte infiltration, and potential skewing toward alternatively activated macrophages—combined with RIDD-mediated cytokine mRNA degradation, may collectively explain the uncoupling of TLR4 engagement from inflammatory cytokine production.

### Proposed Mechanistic Model

**Figure 6:**
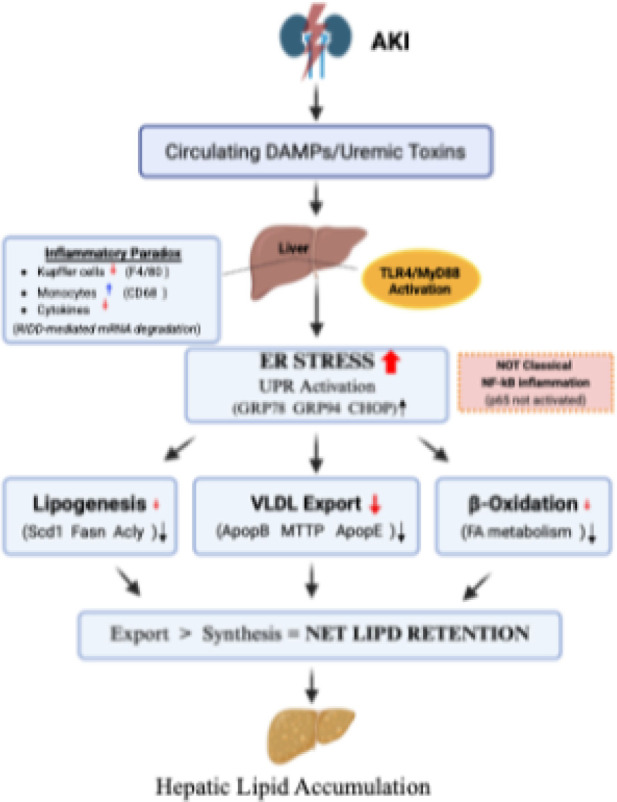
Proposed Mechanistic Model of AKI-Induced Hepatic Steatosis Schematic illustration of the proposed mechanism linking acute kidney injury to hepatic steatosis. AKI triggers release of circulating DAMPs and uremic toxins that engage the hepatic TLR4/MyD88 signaling complex. Rather than inducing classical NF-κB-dependent inflammation, this activation precipitates ER stress with robust UPR activation (evidenced by elevated GRP78, GRP94, CHOP, and XBP1 splicing). ER stress impairs VLDL assembly and secretion machinery (ApoB, MTTP, ApoE downregulation), while lipogenesis and β-oxidation pathways are also suppressed as part of a broader metabolic shutdown. Critically, the magnitude of export failure exceeds synthesis reduction, resulting in net hepatic lipid retention and steatosis. The paradoxical decrease in inflammatory cytokines despite TLR4 engagement reflects Kupffer cell depletion (F4/80↓), monocyte infiltration (CD68↑), and likely RIDD-mediated mRNA degradation.

Based on these findings, we propose the following mechanism for AKI-induced hepatic steatosis (Fig. 6). Acute kidney injury releases circulating DAMPs and uremic toxins that engage the hepatic TLR4 signaling complex. Rather than triggering classical NF-κB-dependent inflammation, this activation induces ER stress, as demonstrated by robust UPR activation concurrent with suppressed NF-κB pathway activity. ER stress impairs VLDL assembly and secretion machinery, evidenced by coordinated downregulation of ApoB, MTTP, and ApoE. Although lipogenesis and β-oxidation are also suppressed—likely representing a broader metabolic shutdown—the magnitude of export failure exceeds synthesis reduction, resulting in net hepatic lipid retention and steatosis. The paradoxical decrease in inflammatory cytokines despite TLR4 engagement reflects both Kupffer cell depletion and RIDD-mediated degradation of cytokine mRNAs, consistent with the observed dissociation between elevated TNF pathway activity and suppressed TNF-α transcript levels.

Schematic illustration of the proposed mechanism linking acute kidney injury to hepatic steatosis. AKI triggers release of circulating DAMPs and uremic toxins that engage the hepatic TLR4/MyD88 signaling complex. Rather than inducing classical NF-κB-dependent inflammation, this activation precipitates ER stress with robust UPR activation (evidenced by elevated GRP78, GRP94, CHOP, and XBP1 splicing). ER stress impairs VLDL assembly and secretion machinery (ApoB, MTTP, ApoE downregulation), while lipogenesis and β-oxidation pathways are also suppressed as part of a broader metabolic shutdown. Critically, the magnitude of export failure exceeds synthesis reduction, resulting in net hepatic lipid retention and steatosis. The paradoxical decrease in inflammatory cytokines despite TLR4 engagement reflects Kupffer cell depletion (F4/80↓), monocyte infiltration (CD68↑), and likely RIDD-mediated mRNA degradation.

### Clinical Validation: AKI Induces Hepatic Dysfunction and Lipid Derangements in ICU Patients

**Figure 7:**
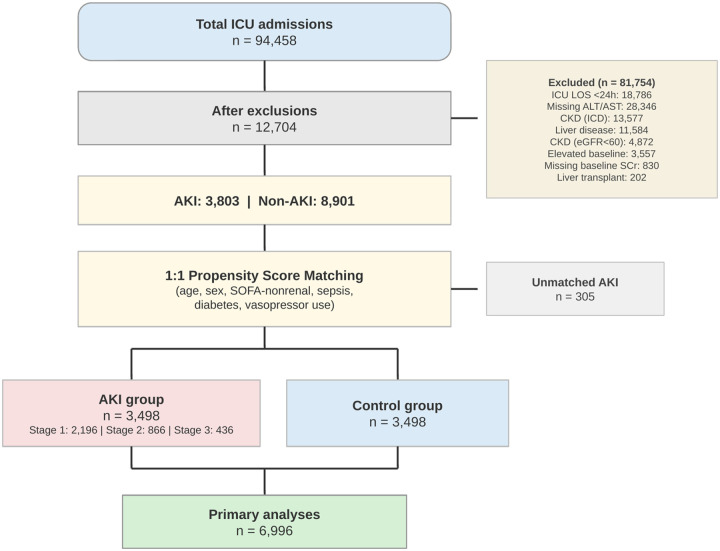
Clinical Validation of AKI-Associated Hepatic Injury in MIMIC-IV ICU Patients CONSORT-style flowchart depicting cohort derivation and propensity score matching (1:1 nearest-neighbor, caliper = 0.2 SD of logit propensity score) from MIMIC-IV version 3.1. The final analytic cohort comprised 3,498 AKI and 3,498 matched control ICU admissions.

**Figure 8:**
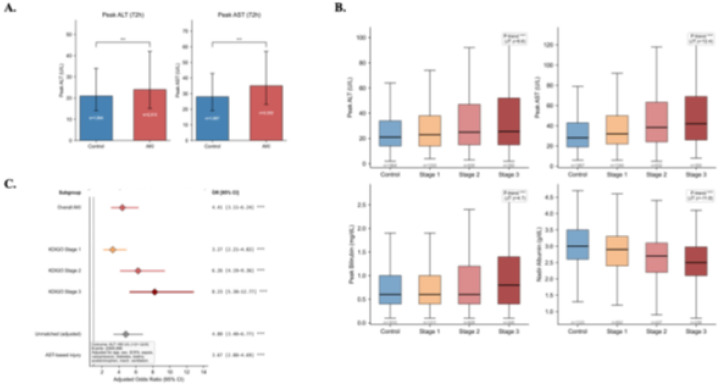
AKI Severity Is Associated with Dose-Dependent Hepatic Injury in ICU Patients (A) Peak serum aminotransferase levels (ALT, left; AST, right) within 72 hours of AKI onset. Box plots display median and interquartile range. ***p < 0.001 by Mann-Whitney U test. (B) Dose-response relationship between AKI severity (KDIGO stages 1, 2, and 3) and liver markers (ALT, AST, total bilirubin, albumin). Values are median with interquartile range. (C) Temporal trajectories of liver function markers from 48 hours before through 168 hours after AKI onset, estimated by mixed-effects models. Shaded regions represent 95% confidence bands. Time-by-group interactions were significant for ALT, AST, and bilirubin (all p < 0.001).

**Figure 9:**
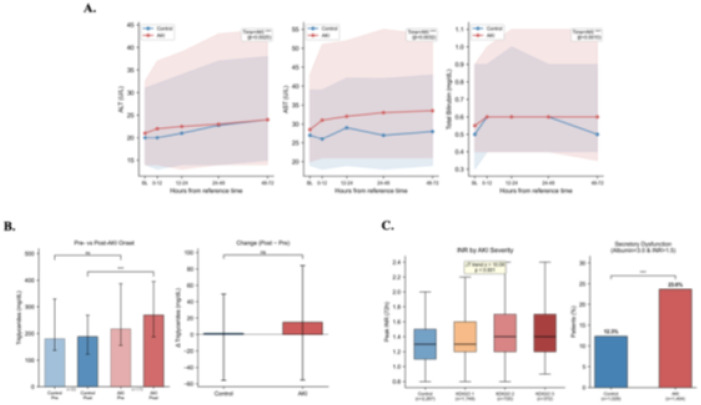
AKI Independently Predicts Liver Injury and Is Associated with Elevated Serum Triglycerides (A) Forest plot of adjusted odds ratios for clinically significant liver injury (defined as peak ALT > 80 U/L) overall and stratified by KDIGO stage. Models adjusted for age, sex, SOFA score, sepsis status, vasopressor use, and mechanical ventilation. Error bars indicate 95% confidence intervals. (B) Serum triglyceride concentrations in the subset of patients with available lipid data (n = 230). Post-AKI triglycerides were significantly higher than in matched controls (269 vs 188 mg/dL, p < 0.001). (C) Hepatic secretory function assessed by INR and serum albumin trajectories following AKI onset. AKI patients exhibited reduced albumin nadir and prolonged INR elevation relative to matched controls.

To determine whether AKI-induced hepatic injury extends to the clinical setting, we analyzed 6,996 propensity-matched ICU admissions from MIMIC-IV (3,498 AKI, 3,498 controls; maximum standardized mean difference = 0.037) (Fig. 7, Table 3). AKI patients exhibited significantly higher peak aminotransferases within 72 hours of ICU admission. Median peak ALT was 24 U/L (IQR 15–42) in the AKI group compared with 21 U/L (IQR 14–34) in controls (p < 0.001), and median peak AST was 35 U/L (IQR 23–57) versus 28 U/L (IQR 19–43; p < 0.001) (Fig. 8A). Albumin nadir was also lower in AKI patients (median 2.7 g/dL [IQR 2.3–3.2] versus 3.0 g/dL [IQR 2.6–3.5]; p < 0.001). Liver marker elevations followed a severity-dependent gradient across KDIGO stages (Fig. 8B), and the incidence of clinically significant liver injury (peak ALT > 80 U/L) reached 5.3% in the AKI group compared with 1.2% in controls. In multivariable logistic regression adjusting for age, sex, SOFA score, sepsis, vasopressor use, and mechanical ventilation, the adjusted odds ratio for liver injury was 4.41 (95% CI: 3.11–6.24; p < 0.001) overall, with a graded increase by KDIGO stage: 3.27 for stage 1, 6.26 for stage 2, and 8.23 for stage 3 (Fig. 8C). Mixed-effects modeling confirmed progressive divergence of liver markers after AKI onset, with significant time-by-group interactions for ALT, AST, and bilirubin (all p < 0.001) (Fig. 9A). In an exploratory analysis of patients with available triglyceride data (n = 230), post-AKI triglycerides were higher than in controls (269 versus 188 mg/dL; p < 0.001) (Fig. 9B). Peak INR also demonstrated a severity-dependent increase across KDIGO stages (Jonckheere-Terpstra z = 10.04, p < 0.001), and a composite measure of hepatic secretory dysfunction (albumin nadir < 3.0 g/dL and peak INR > 1.5) was nearly twice as prevalent in AKI patients as in controls (23.6% versus 12.3%; p < 0.001) (Fig. 9C). The AKI-liver injury association was independent of sepsis status (interaction p = 0.364) and persisted across multiple sensitivity analyses.

Analysis of lipid profiles in 22,727 ICU patients from the eICU-CRD, a multi-center cohort spanning 208 hospitals (5,956 AKI, 16,771 non-AKI), revealed a lipid dissociation pattern in AKI patients: serum triglycerides were elevated (median 124 [IQR: 85–192] versus 111 [IQR:78–162] mg/dL; p = 4.9 × 10^−40^), while total cholesterol (131 versus 148 mg/dL; p = 6.3 × 10^−62^), HDL (35 versus 39 mg/dL; p = 2.0 × 10^−49^), and LDL (69 versus 81 mg/dL; p = 1.2 × 10^−48^) were all decreased. After adjustment for age, sex, APACHE IV score, BMI, and diabetes, the triglyceride elevation persisted (adjusted β = +0.076, corresponding to 7.9% increase; p = 9.1 × 10^−16^). Triglyceride levels scaled with AKI severity in a dose-dependent fashion, from + 3.7% in KDIGO stage 1 to + 18.4% in stage 3 (Kruskal-Wallis p = 2.7 × 10^−68^). Temporal analysis showed that triglycerides were elevated in AKI patients at every measured time window from admission through day 7 (mixed-effects model: AKI β = +0.118, p = 2.7 × 10^−8^), with no significant time-by-AKI interaction (p = 0.30). The elevation was present in all examined subgroups, including sepsis and non-sepsis, surgical and medical admissions, and diabetic and non-diabetic patients. Both databases independently demonstrated elevated triglycerides in AKI patients (Supplementary Figure S4).

## Discussion

The liver's vulnerability to AKI-induced remote injury is well established, with prior studies attributing damage primarily to inflammatory cascades and oxidative stress^1,6^. However, the metabolic consequences have remained surprisingly under-explored. The central finding of this study is that AKI-induced hepatic steatosis arises primarily from impaired lipid export rather than enhanced lipogenesis, a mechanism fundamentally distinct from classical fatty liver disease models. In conventional steatosis paradigms, hepatic triglyceride accumulation reflects increased fatty acid uptake, upregulated de novo lipogenesis, or impaired β-oxidation^25,26^. Our proteomics data reveal an unexpected paradox: despite pronounced lipid accumulation, all major lipid metabolic pathways were uniformly suppressed. Lipogenic enzymes (Scd1, Fasn, Acly) were downregulated, and fatty acid oxidation machinery was similarly diminished. These observations argue against enhanced synthesis as the driver and instead point toward impaired clearance. We demonstrate that ER stress-mediated disruption of VLDL assembly provides the critical mechanistic link. A previous study showed that renal ischemia-reperfusion induces fatty liver through VLDL-triglyceride secretion inhibition^27^, supporting export failure as a relevant mechanism in kidney-liver crosstalk. Our data extend this framework by identifying ER stress as the bridge linking AKI-derived signals to hepatic lipid retention.

Our retrospective analysis of nearly 7,000 propensity-matched ICU patients from MIMIC-IV provides clinical support for these experimental observations. AKI was independently associated with elevated aminotransferases and a fourfold increase in clinically meaningful liver injury (adjusted OR = 4.41; 95% CI: 3.11–6.24), an association that followed a severity-dependent gradient across KDIGO stages. Liver markers diverged progressively from matched controls after AKI onset, consistent with hepatic injury arising secondary to the renal insult. That this relationship persisted after adjustment for sepsis, organ failure severity, and hepatotoxic exposures is particularly relevant, given that our murine model employs sterile ischemia-reperfusion rather than infection-driven AKI. Several prior MIMIC-based studies have reported associations between AKI and hepatic dysfunction in specific populations such as cardiac surgery or sepsis patients; our analysis extends this work by demonstrating the relationship across a broad ICU population after excluding patients with pre-existing liver or kidney disease. Beyond aminotransferases, peak INR showed a severity-dependent increase across KDIGO stages, and the prevalence of combined hepatic secretory dysfunction—defined as concurrent hypoalbuminemia and INR prolongation—was nearly doubled in AKI patients. Because albumin and coagulation factors are synthesized through ER-dependent secretory pathways, these findings parallel the impaired ApoB/VLDL export identified in our proteomic analysis and suggest a broad defect in hepatic secretory function consistent with ER stress.

The lipid dissociation pattern identified in the eICU-CRD cohort complicates a simple export failure model. While decreased total cholesterol, HDL, and LDL are consistent with impaired hepatic lipoprotein assembly, as predicted by ApoB and MTTP downregulation, the concurrent elevation of serum triglycerides is not readily explained by export failure alone. This dissociation likely reflects two concurrent processes. Systemic stress during critical illness drives catecholamine-mediated adipose lipolysis, releasing free fatty acids that the liver takes up and re-esterifies^28,29^. Simultaneously, ER stress impairs the assembly of mature VLDL particles, trapping these re-esterified lipids within hepatocytes. The liver retains enzymatic capacity for fatty acid esterification, a process that occurs independently of ER quality control, but cannot package the resulting triglycerides into secretion-competent lipoproteins. Together, these processes produce a dual metabolic insult: the liver receives an increased lipid load from peripheral sources but cannot efficiently export it. Suppression of lipoprotein lipase activity by pro-inflammatory cytokines may further contribute to elevated circulating triglycerides by reducing peripheral clearance^28,30,31^. That both MIMIC-IV and eICU-CRD independently demonstrated triglyceride elevation in AKI patients, with dose-dependent scaling across KDIGO stages, argues against this pattern reflecting confounding by overall illness severity.

The proteomics findings challenge conventional assumptions about hepatic steatosis. Key lipogenic enzymes—Scd1 (log2FC = − 1.59), Fasn, Acly, and Acaca—were uniformly suppressed, as was fatty acid oxidation machinery including Cpt1a and acyl-CoA dehydrogenases. This broad metabolic shutdown likely reflects the catabolic state of acute illness but fails to explain triglyceride accumulation. The resolution lies in relative imbalance. Hepatic triglyceride homeostasis requires coordination among four processes: fatty acid uptake, de novo lipogenesis, β-oxidation, and VLDL-mediated export^9^. When we examined export machinery, ApoB (log2FC = − 0.23, adj. p = 0.005), MTTP (log2FC = − 0.25, adj. p = 0.022), and ApoE (log2FC = − 0.56) were all significantly reduced. These proteins are essential for VLDL assembly—ApoB serves as the structural scaffold, MTTP facilitates lipid loading onto nascent particles, and ApoE aids secretion and hepatic clearance^32^. Their coordinated downregulation indicates that while the liver reduces lipid synthesis in response to acute injury, it reduces export capacity even more severely. The magnitude of export failure exceeds synthesis reduction, resulting in net lipid retention. Notably, while increased hepatic triglyceride content is the hallmark of steatosis, accumulation of free fatty acids (FFA) and total cholesterol (TC) may be more directly hepatotoxic, as FFA and cholesterol overload can trigger lipotoxic injury through oxidative stress and mitochondrial dysfunction. Whether AKI alters hepatic FFA and TC levels independently of triglyceride accumulation remains to be determined and warrants direct measurement in future studies. Our proteomic dataset did not capture several proteins involved in hepatic fatty acid uptake (e.g., CD36, LDLR), intracellular lipolysis (e.g., ATGL/PNPLA2, HSL/LIPE, MGL/MGLL, ABHD5/CGI-58), or lipid droplet stabilization (e.g., perilipin family members, CIDEA), likely reflecting the inherent bias of untargeted proteomics toward abundant proteins. Targeted studies of these pathways would clarify the relative contributions of lipid uptake, intracellular remodeling, and droplet dynamics to AKI-induced steatosis. As described above, clinical lipid profiles in AKI patients suggest that enhanced peripheral lipid delivery further amplifies this imbalance.

The connection between ER stress and impaired VLDL secretion has substantial mechanistic precedent. ApoB (~ 550 kDa) requires extensive cotranslational lipidation within the ER lumen—a process exquisitely sensitive to ER homeostasis perturbations^2,33^. Under ER stress, the unfolded protein response activates quality control mechanisms promoting degradation of misfolded proteins through ER-associated degradation (ERAD); ApoB is particularly vulnerable to this pathway^11,34^. We documented robust UPR activation through multiple lines of evidence: proteomics revealed upregulation of ER chaperones—GRP78/BiP (log2FC = + 0.36), GRP94 (log2FC = + 0.42), and p58IPK (log2FC = + 0.43)—along with the translocon component Sec61α. Western blotting confirmed elevated GRP78 and CHOP protein levels, and XBP1 splicing was markedly increased, demonstrating functional IRE1α engagement. The concurrent UPR activation and reduced ApoB/MTTP abundance strongly support a model wherein ER stress impairs VLDL assembly machinery, trapping triglycerides within hepatocytes. Notably, this differs from lipotoxicity-induced ER stress in NAFLD, where excess lipid accumulation secondarily triggers ER dysfunction^14^. In AKI, the temporal sequence appears reversed: ER stress precedes and causes lipid retention. Beyond its role in impairing VLDL assembly, prolonged ER stress activates cell death signaling through multiple downstream effectors. CHOP upregulation, as documented in our data, can initiate apoptosis via transcriptional induction of pro-apoptotic targets. In parallel, IRE1α-mediated activation of JNK and downstream NF-κB signaling, as well as caspase-3/7 activation through the mitochondrial apoptotic pathway, represent additional ER stress-induced cell death mechanisms that may contribute to hepatocyte injury during AKI. Whether these pathways are functionally engaged in our model requires further investigation using pathway-specific inhibitors or activity assays.

An unexpected finding was the dissociation between TLR4/MyD88 engagement and classical inflammatory output. TLR4 signaling typically drives robust NF-κB-dependent cytokine transcription^13^. We found TLR4 pathway priming—CD14 transcripts were elevated—yet NF-κB activity remained unchanged by PROGENy analysis and pro-inflammatory cytokines (TNF-α, IL-1β, IL-6) were paradoxically reduced at both transcript and protein levels. Several mechanisms may explain this dissociation: activated IRE1α can degrade cytokine mRNAs through regulated IRE1-dependent decay (RIDD)^35^, and prior DAMP exposure during AKI may induce endotoxin tolerance^36^. Most compellingly, the hepatic macrophage landscape underwent substantial remodeling. F4/80-positive Kupffer cells—the liver's primary cytokine source—were significantly depleted, while CD68-positive monocyte-derived macrophages increased. CD163 upregulation suggests the infiltrating population exhibits an alternatively activated phenotype. Kupffer cell loss directly accounts for reduced cytokine output regardless of TLR4 signaling status, removing the cellular machinery required for classical inflammatory responses.

These findings identify potential therapeutic targets for preventing hepatic dysfunction during AKI. ER stress modulation using chemical chaperones such as 4-phenylbutyric acid (4-PBA) or tauroursodeoxycholic acid (TUDCA) represents an attractive strategy, with demonstrated hepatoprotective effects in ER stress-induced liver injury models^37,38^. Kim and colleagues showed that taurine supplementation normalized MTTP and ApoB expression while reducing ER stress markers^37^—directly relevant to our proposed mechanism. The timing of intervention appears critical, as our 24-hour timepoint likely captures an early window when ER stress is actively disrupting VLDL machinery. Several limitations warrant acknowledgment. Our single timepoint precludes assessment of temporal dynamics —whether ER stress precedes lipid accumulation cannot be definitively established without time-course studies. However, the concurrent upregulation of early UPR markers (GRP78, XBP1 splicing) alongside only modest triglyceride accumulation at 24 hours is consistent with ER stress initiating prior to full steatosis development; formal time-course experiments will be needed to confirm this sequence. We studied only male mice, leaving sex differences unexplored; Yang and colleagues demonstrated significant sex disparity in NAFLD related to estrogen-receptor-α-dependent VLDL secretion^39^. While proteomics provides comprehensive abundance data, it does not directly measure metabolic flux. Importantly, triglyceride measurements provide direct functional validation of the export failure model: plasma triglyceride levels were significantly reduced in AKI mice, consistent with diminished VLDL secretion, while liver tissue triglyceride content was correspondingly elevated (Fig. 4D). This reciprocal pattern strongly supports impaired VLDL-mediated export as the primary driver of hepatic lipid retention. Most importantly, establishing causation would require demonstrating that chemical chaperones prevent both ER stress and steatosis. The clinical analyses presented here represent an initial step toward such translation, although the retrospective, observational design of the MIMIC-IV and eICU-CRD studies precludes causal inference. Serum aminotransferase elevations in critically ill patients may reflect hepatic ischemia, drug-induced injury, or systemic inflammation rather than the specific lipotoxic mechanism we propose. The triglyceride and lipoprotein data cannot distinguish hepatic from extrahepatic contributions to circulating lipid changes, and absolute triglyceride values differed between the two databases (median 269 mg/dL in MIMIC-IV vs. 124 mg/dL in eICU-CRD), likely reflecting differences in measurement timing and cohort composition. Still, concordant directionality across two independent databases with distinct populations and ascertainment methods, combined with dose-dependent relationships to AKI severity, supports the clinical relevance of these findings. Future studies should incorporate pharmacological ER stress intervention, direct VLDL secretion rate measurements, and detailed lipid profiling with serial measurements in prospectively enrolled AKI patients.

## Conclusions

This study establishes that AKI-induced hepatic steatosis arises primarily from ER stress-mediated impairment of VLDL export machinery rather than enhanced lipogenesis. The discovery of global metabolic suppression concurrent with disproportionate export failure resolves the paradox of steatosis in a catabolic state. Clinical lipid profiles from two independent ICU databases corroborate these experimental findings and reveal a dual metabolic insult in which impaired hepatic lipoprotein export is compounded by enhanced peripheral lipid delivery during critical illness. These integrated experimental and clinical observations support targeting hepatic ER stress pathways as a strategy for mitigating remote liver injury during AKI, distinct from anti-inflammatory interventions that have dominated the field.

## Supplementary Material

Supplementary Files

This is a list of supplementary files associated with this preprint. Click to download.

- SupplementalMaterials.pdf

## Figures and Tables

**Table 1:** Key differentially expressed proteins in hepatic ER stress and lipid metabolism pathways

Functional Category	Protein (Gene)	log_2_FC	Adj. P	Biological Function
ER Stress / UPR	GRP78 (Hspa5)	+ 0.36	0.052	ER chaperone, UPR master regulator
	GRP94 (Hsp90b1)	+ 0.42	0.036	ER chaperone, protein quality control
	p58IPK (Dnajc3)	+ 0.43	0.018	Co-chaperone, UPR modulator
	Sec61α (Sec61a1)	+ 0.36	0.021	ER translocon component
Lipid Export	ApoB (Apob)	−0.23	0.005	VLDL structural protein
	MTTP (Mttp)	−0.25	0.022	Lipid transfer to ApoB
	ApoE (Apoe)	−0.56	0.072	Lipoprotein component
Lipogenesis	SCD1 (Scd1)	−1.59	0.031	Fatty acid desaturation
	FASN (Fasn)	−0.59	0.151	Fatty acid synthesis
	ACLY (Acly)	−0.41	0.258	Citrate to acetyl-CoA conversion

FC, fold change; UPR, unfolded protein response; VLDL, very low-density lipoprotein. P-values adjusted using Benjamini-Hochberg method. Positive FC indicates upregulation in AKI vs. Sham.

**Table 2:** Hepatic lipid metabolism proteins altered following acute kidney injury

Functional Category	Protein	Gene	log_2_FC	Adj. P	Biological Function
De novo Lipogenesis	SCD1	Scd1	−1.59	0.031	Fatty acid desaturase (ratelimiting)
	FASN	Fasn	−0.59	0.151	Fatty acid synthase
	ACLY	Acly	−0.41	0.258	ATP-citrate lyase
	ACC	Acaca	−0.67	NS	Acetyl-CoA carboxylase α
β-Oxidation	CPT1A	Cpt1a	−0.35	< 0.05	Carnitine palmitoyltransferase 1A
	CPT2	Cpt2	−0.28	NS	Carnitine palmitoyltransferase 2
	ACADL	Acadl	−0.42	NS	Long-chain acyl-CoA dehydrogenase
	ACADM	Acadm	−0.31	NS	Medium-chain acyl-CoA dehydrogenase
	ACADVL	Acadvl	−0.26	NS	Very long-chain acyl-CoA dehydrogenase
	HADHA	Hadha	−0.38	NS	Trifunctional protein α subunit
	HADHB	Hadhb	−0.29	NS	Trifunctional protein β subunit
	ECHS1	Echs1	−0.33	NS	Enoyl-CoA hydratase
	ACAT1	Acat1	−0.25	NS	Acetyl-CoA acetyltransferase 1
VLDL Assembly/Export	ApoB	Apob	−0.23	0.005	VLDL structural apolipoprotein
	MTTP	Mttp	−0.25	0.022	Microsomal triglyceride transfer protein
	ApoE	Apoe	−0.56	0.072	Apolipoprotein E

FC, fold change; NS, not significant (adj. P > 0.05). P-values adjusted using Benjamini-Hochberg method. All proteins showed decreased abundance in AKI versus Sham livers. Note the consistent directional change (suppression) across all three functional categories, with VLDL export proteins showing the most consistent statistical significance despite modest fold changes.

**Table 3:** Baseline characteristics of propensity score-matched AKI and non-AKI ICU cohorts (MIMIC-IV)

Variable	Overall (N = 6,996)	AKI (n = 3,498)	Control (n = 3,498)	SMD	P-Value
Age (years), mean (SD)	63.4 (16.1)	63.1 (16.1)	63.7 (16.1)	0.037	0.121
Male sex, n (%)	3,850 (55.0)	1,933 (55.3)	1,917 (54.8)	0.009	0.718
Race/Ethnicity, n (%)				0.044	0.638
White	4,493 (64.2)	2,230 (63.8)	2,263 (64.7)		
Black	669 (9.6)	356 (10.2)	313 (8.9)		
Hispanic/Latino	264 (3.8)	127 (3.6)	137 (3.9)		
Asian	224 (3.2)	112 (3.2)	112 (3.2)		
Other	324 (4.6)	163 (4.7)	161 (4.6)		
Unknown	1,022 (14.6)	510 (14.6)	512 (14.6)		
Admission category, n (%)				0.038	0.466
Elective	180 (2.6)	86 (2.5)	94 (2.7)		
Emergency	1,481 (21.2)	765 (21.9)	716 (20.5)		
Other	4,983 (71.2)	2,477 (70.8)	2,506 (71.6)		
Surgical	352 (5.0)	170 (4.9)	182 (5.2)		
SOFA score (total), median [Q1, Q3]	4.0 [3.0, 7.0]	5.0 [3.0, 7.0]	4.0 [3.0, 7.0]	0.110	< 0.001
SOFA score (non-renal), median [Q1, Q3]	4.0 [2.0, 6.0]	4.0 [2.0, 6.0]	4.0 [2.0, 6.0]	0.010	0.417
Sepsis, n (%)	3,342 (47.8)	1,657 (47.4)	1,685 (48.2)	0.016	0.518
Diabetes, n (%)	1,965 (28.1)	1,001 (28.6)	964 (27.6)	0.024	0.338
Vasopressor use, n (%)	2,965 (42.4)	1,494 (42.7)	1,471 (42.1)	0.013	0.595
Baseline ALT (U/L), median [Q1, Q3]	21.0 [14.0, 32.0]	21.0 [14.0, 33.0]	20.0 [14.0, 31.0]	0.091	0.013
Baseline AST (U/L), median [Q1, Q3]	28.0 [19.0, 41.0]	29.0 [20.0, 44.0]	26.0 [19.0, 39.0]	0.147	< 0.001
ICU LOS (hours), median [Q1, Q3]	82.0 [47.0, 165.0]	116.0 [60.0, 243.8]	64.5 [42.0, 110.0]	0.595	< 0.001
Hospital mortality, n (%)	1,043 (14.9)	710 (20.3)	333 (9.5)	0.306	< 0.001

AKI, acute kidney injury; ALT, alanine aminotransferase; AST, aspartate aminotransferase; ICU, intensive care unit; LOS, length of stay; SD, standard deviation; SMD, standardized mean difference; SOFA, Sequential Organ Failure Assessment. Propensity score matching was performed on age, sex, race/ethnicity, admission category, non-renal SOFA score, sepsis, diabetes, and vasopressor use. SMD < 0.1 indicates adequate balance.

## Data Availability

The proteomics dataset generated in this study has been deposited in Mendeley Data and is publicly available at https://doi.org/10.17632/kvm2f3fvxg.1. Clinical data were obtained from the Medical Information Mart for Intensive Care IV (MIMIC-IV, version 3.1) database, available through PhysioNet (https://physionet.org/content/mimiciv/3.1/), and the eICU Collaborative Research Database (eICU-CRD), available through PhysioNet (https://physionet.org/content/eicu-crd/), both under signed data use agreements[18, 21]. All analysis code for the clinical validation component and all other data supporting the findings of this study are available from the corresponding author upon reasonable request.

## Abbreviations

4-PBA: 4-phenylbutyric acid
AKI: acute kidney injury
ALT: alanine aminotransferase
APACHE IV: Acute Physiology and Chronic Health Evaluation IV
ApoB: apolipoprotein B
ApoE: apolipoprotein E
AST: aspartate aminotransferase
BiP: binding immunoglobulin protein
BMI: body mass index
BUN: blood urea nitrogen
CHOP: C/EBP homologous protein
CI: confidence interval
DAMPs: damage-associated molecular patterns
DEqMS: differential expression analysis using quality-weighted minimum p-values and sums
eICU-CRD: eICU Collaborative Research Database
ER: endoplasmic reticulum
ERAD: ER-associated degradation
FDR: false discovery rate
GFR: glomerular filtration rate
GRP78: glucose-regulated protein 78
GRP94: glucose-regulated protein 94
GSEA: gene set enrichment analysis
H&E: hematoxylin and eosin
HDL: high-density lipoprotein
ICU: intensive care unit
IHC: immunohistochemistry
IL-1β: interleukin-1 beta
IL-6: , interleukin-6
INR: international normalized ratio
IQR: interquartile range
IR: ischemia-reperfusion
IRE1α: inositol-requiring enzyme 1 alpha
IRI: ischemia-reperfusion injury
KDIGO: Kidney Disease: Improving Global Outcomes
KIM-1: kidney injury molecule-1
LC-MS: liquid chromatography-mass spectrometry
LDL: low-density lipoprotein
log_2_FC: log_2_ fold-change
MIMIC-IV: Medical Information Mart for Intensive Care IV
MTTP: microsomal triglyceride transfer protein
mTORC1: mechanistic target of rapamycin complex 1
NAFLD: non-alcoholic fatty liver disease
NES: normalized enrichment score
NF-κB: nuclear factor kappa-light-chain-enhancer of activated B cells
NGAL: neutrophil gelatinase-associated lipocalin
OR: odds ratio
PCA: principal component analysis
PDI: protein disulfide isomerase
PERK: protein kinase R-like endoplasmic reticulum kinase
PPAR: peroxisome proliferator-activated receptor
qPCR: quantitative polymerase chain reaction
RIDD: regulated IRE1-dependent decay
SEM: standard error of the mean
SOFA: Sequential Organ Failure Assessment
TC: total cholesterol
TG: triglycerides
TLR4: Toll-like receptor 4
TNF-α: tumor necrosis factor alpha
TUDCA: tauroursodeoxycholic acid
UPR: unfolded protein response
VLDL: very-low-density lipoprotein
XBP1: X-box binding protein 1
XBP1s: spliced X-box binding protein 1
